# Added Effects of Loaded Sit-to-Stand Exercise on Sit-to-Stand Ability and Gait Speed in Stroke: An Experimental Study

**DOI:** 10.7759/cureus.107505

**Published:** 2026-04-21

**Authors:** Aayushi Shah, Priti Agni

**Affiliations:** 1 Neurophysiotherapy, K J Somaiya College of Physiotherapy, Mumbai, IND

**Keywords:** functional strength training, gait speed, neurorehabilitation, post-stroke mobility, sit-to-stand training, stroke rehabilitation

## Abstract

Introduction: Stroke frequently results in motor impairments that affect one side of the body, leading to difficulty in performing everyday functional tasks. Among these, sit-to-stand (STS) movement and walking are commonly affected due to reduced lower limb strength, impaired balance, and asymmetrical weight-bearing. The STS movement is a fundamental transitional task required for independence, as it bridges static sitting to dynamic mobility. Since STS performance shares biomechanical and muscular demands with walking, targeted strengthening during this task may influence gait performance. Therefore, this study aimed to determine the added effects of loaded STS exercise on STS ability and gait speed in individuals with stroke.

Materials and methods: This experimental study was conducted on 34 stroke participants recruited from the physiotherapy outpatient department of a tertiary care hospital. Participants were screened based on the inclusion and exclusion criteria and randomly allocated into two equal groups. Group A (n = 17) received loaded STS exercise along with conventional therapy, while Group B (n = 17) received only conventional therapy. Pre-intervention assessment was performed using the STS test via NeuroCom Balance Master (NeuroCom International, Inc., Clackamas, OR) to assess STS ability parameters, including weight transfer time, rising index, weight-bearing symmetry, and center of gravity sway velocity. Gait speed was assessed using the 10-meter walk test. Both groups received treatment for 60 minutes per session, three times per week for two weeks. Post-intervention assessments were performed at the end of the second week using the same outcome measures.

Results: Intragroup analysis showed a statistically significant difference between pre- and post-intervention values for STS ability parameters, including weight transfer time, rising index, weight-bearing symmetry, and center of gravity sway velocity, in both groups (p < 0.05). Gait speed assessed using the 10-meter walk test also showed statistically significant improvement within both groups. However, intergroup analysis revealed statistically significant differences between Groups A and B for STS ability parameters and gait speed, indicating greater improvement in the experimental group receiving loaded STS exercise.

Conclusion: Loaded STS exercise, along with conventional therapy, significantly improved STS ability and gait speed in stroke patients. Compared to conventional therapy alone, loaded STS exercise demonstrated greater improvement in STS parameters and walking speed.

## Introduction

Stroke is a leading cause of disability and mortality globally, with over 13 million new cases annually. According to the Global Burden of Disease study, stroke ranks third in disability and mortality worldwide and is also the fifth leading cause of morbidity, accounting for 3.5% of disability-adjusted life years (DALYs). There is an increasing burden of stroke in India, making it the fourth leading cause of death and the fifth leading cause of disability. The incidence of stroke in India is estimated to range between 108 and 172 cases per 100,000 population per year [1].

The main deficit caused by stroke is motor impairment that affects the control of movement of the face, arm, and leg on one side of the body and is present in about 80% of patients. Almost two-thirds of stroke survivors have initial mobility deficits, and many continue to face difficulty in walking independently [2]. These mobility limitations arise due to factors such as asymmetry of stride time and length, reduced walking velocity, poor joint and postural control, muscle weakness, and abnormal muscle activation patterns following stroke [3]. As a result, individuals with stroke often face challenges while performing functional transitions such as supine-to-sit, sit-to-stand (STS), and other inter-surface transfers. The inability to perform STS independently can prevent independent functioning during activities of daily living (ADLs) [4].

STS movement is a crucial functional motor task performed repeatedly during daily activities. Successful execution of this movement requires coordinated activity of the lower limbs to transfer body weight from sitting to standing while maintaining balance and stability. Stroke patients frequently experience balance loss while standing due to impaired postural control, weakened muscles, and unsteady movements [5]. The STS movement demands adequate lower limb muscle strength, symmetrical weight-bearing, and effective postural control to transfer the center of mass within a limited base of support.

An efficient standing ability precedes normal gait. Stroke-induced upper motor neuron syndrome leads to several sensorimotor impairments, including muscle weakness, spasticity, altered proprioception, and impaired motor control, which affect the normal walking pattern [6]. Reduced walking speed is a characteristic feature of post-stroke gait; the normal walking speed is approximately 1.3 m/s in healthy individuals, whereas it ranges between 0.23 and 0.73 m/s in individuals with stroke [2,3]. STS movement acts as a bridge between static posture and dynamic activities such as walking. Both STS and walking involve coordinated lower extremity movements, weight transfer, balance control, and stability.

Task-specific repetitive STS training has been shown to improve loading on the paretic limb and enhance lower limb muscle force generation, thereby improving strength and dynamic stability in stroke patients [7,8]. Progressive resistance training for the lower extremities has also been shown to improve gait parameters, particularly walking speed and walking distance in stroke patients [9]. However, there is limited evidence in the literature regarding whether task-specific progressive strength training applied during STS activities significantly improves various parameters of STS ability and gait speed in stroke patients. Previous studies have reported that loaded STS exercise training improves functional performance, such as walking and balance, in individuals with stroke, but limited research has examined its effect on specific STS parameters and gait outcomes [10]. Thus, the purpose of this study is to determine the added effects of loaded STS exercise on STS ability and gait speed in stroke patients.

## Materials and methods

This study was conducted over a period of 18 months among stroke participants recruited from the physiotherapy outpatient department (OPD) of a tertiary care hospital. Institutional ethics committee approval was obtained prior to the commencement of the study, and written informed consent was obtained from all participants. The study was reported in accordance with the CONSORT (Consolidated Standards of Reporting Trials) guidelines.

Participants were screened based on predefined inclusion and exclusion criteria. Inclusion criteria comprised right or left hemiplegic patients with clinically diagnosed first stroke, who were able to walk 10 m with or without assistance and were able to perform STS without assistance [4]. Exclusion criteria included patients with hemiplegia due to causes other than cerebrovascular accident, such as traumatic brain injury; patients with medical conditions, such as unstable hypertension, heart diseases, or orthopedic problems that could prevent participation in the intervention; and patients who were unable to understand or follow verbal commands [4].

**Figure 1:**
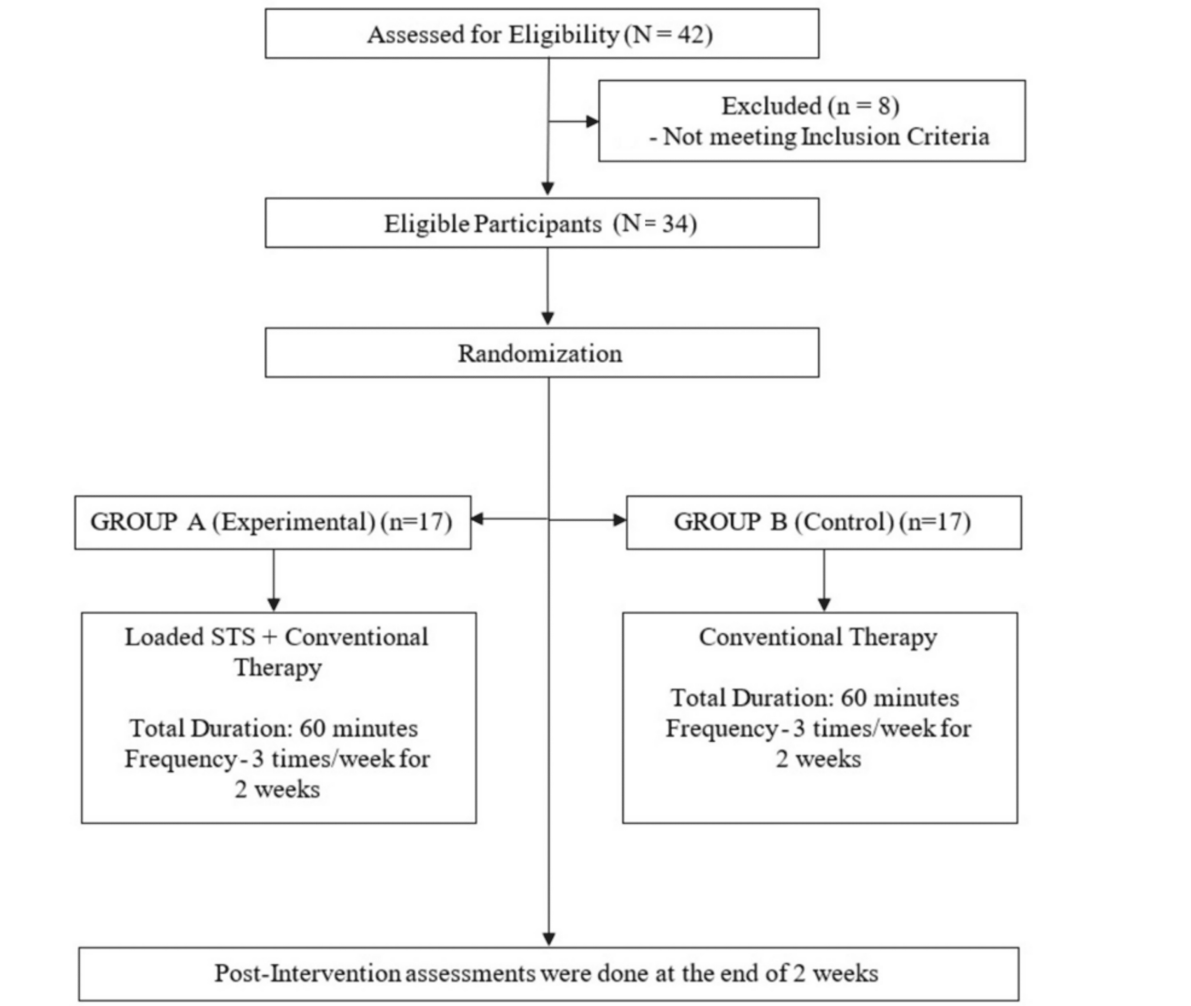
Participant flow diagram STS: Sit-to-stand.

Sample size was estimated based on the clinical prevalence of stroke in the physiotherapy OPD of a tertiary care hospital, which was found to be 77.78%. Using a 95% confidence interval (Z = 1.96) and allowing for a 10% attrition rate, the final sample size was determined to be 34 participants. Participants were equally allocated into two groups, with 17 participants in each group. Figure 1 depicts the participant flow throughout the research process.

Participants were recruited using convenience sampling from the OPD based on the inclusion and exclusion criteria. Participants were then randomly allocated into two groups using computer-generated random numbers through the OpenEPI software. Allocation concealment was performed using the SNOSE (sequentially numbered, opaque, sealed envelopes) method. Participants selected an envelope and were allocated to either Group A (experimental group) or Group B (control group) according to the number assigned.

Pre-intervention assessment included recording demographic details such as age, gender, and side of hemiplegia. STS ability was assessed using the STS test via NeuroCom Balance Master (version 8.6.0; NeuroCom International, Inc., Clackamas, OR), which measures parameters including weight transfer time (seconds), rising index (% body weight), weight-bearing symmetry (% difference between limbs), and center of gravity (COG) sway velocity (degrees/second) [11]. Gait speed was assessed using the 10-meter walk test [12].

Participants in Group A received loaded STS exercise along with conventional therapy, whereas participants in Group B received only conventional therapy. Conventional therapy included approaches such as proprioceptive neuromuscular facilitation (PNF), Bobath approach, and motor relearning program (MRP), focusing on re-educating normal movement patterns and task-specific training. It included exercises such as STS, bridging, and squatting; functional exercises for the upper and lower limbs; balance training exercises such as tandem standing, narrow-base standing, spot marching, single-leg standing, step-ups, obstacle crossing, ball catch and throw in standing; and walking in a figure-of-eight pattern. These exercises were performed with eyes open and closed and on different surfaces as progression. Treadmill walking and cycling were also included.

**Figure 2:**
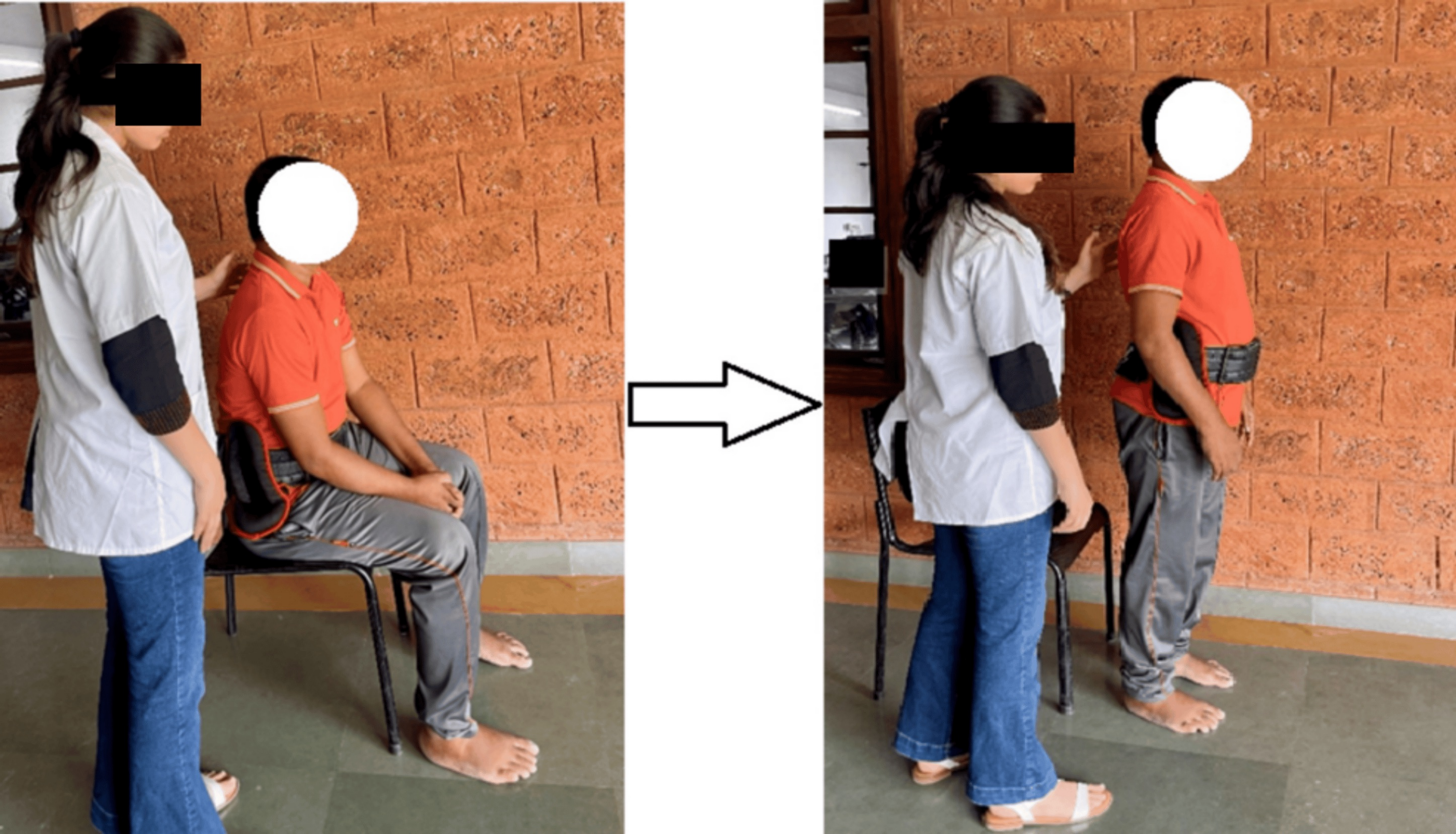
Loaded sit-to-stand exercise

The loaded STS protocol was performed using a weighted belt (Figure 2). One repetition maximum (1-RM) of the loaded STS test was defined as the maximal load a patient could carry while standing up once from a sitting position without loss of balance or assistance [10]. The participant was positioned in a standardized sitting posture with hips and knees flexed, feet placed parallel, and trunk erect. The initial load was selected at a level that allowed the participant to perform the STS task safely and independently. The load was then progressively increased in increments of 5% based on tolerance and successful task performance [13]. A successful attempt was defined as the ability to perform a single STS independently while maintaining stability for five seconds, with adequate rest provided between trials to minimize fatigue. The maximum load at which the participant could successfully complete the task was recorded as the 1-RM.

The protocol consisted of 10 repetitions at 30% of 1-RM followed by two to three minutes of rest, 10 repetitions at 50% of 1-RM followed by two to three minutes of rest, and again 10 repetitions at 30% of 1-RM [10,14].

Both groups received treatment for 60 minutes per session, three times per week for two weeks. Post-intervention assessments were performed at the end of the second week using the same outcome measures.

Data were recorded using Microsoft Excel (Microsoft Corp., Redmond, WA) and analyzed using GraphPad Prism version 9 (GraphPad Software, San Diego, CA). Quantitative variables were expressed as mean ± standard deviation, and qualitative variables were expressed as percentages. The Shapiro-Wilk test was used to assess the normality of data distribution. For normally distributed variables, paired t-tests were used for intragroup comparisons and unpaired t-tests for intergroup comparisons. For variables that did not follow a normal distribution, the Wilcoxon signed-rank test was used for intragroup comparisons and the Mann-Whitney U test for intergroup comparisons. A p-value < 0.05 was considered statistically significant.

## Results

A total of 34 participants were included in the study and completed the intervention. The baseline demographic characteristics of the participants are presented in Table 1.

**Table 1:** Demographic characteristics of the participants M: Male; F: Female; Rt: Right; Lt: Left.

Demographic characteristics	
Age (in years) (Mean ± SD)	51.59 ± 6.51
Gender (M/F in %)	M - 79, F - 21
Side of affection (Rt/Lt in %)	Rt - 56, Lt - 44

Intragroup analysis

In Group A, the paired t-test was used to compare pre- and post-intervention values of weight-bearing symmetry, while the Wilcoxon signed-rank test was used for weight transfer time, rising index, COG sway velocity, and 10-meter walk test (10MWT) scores. In Group B, the paired t-test was used to compare pre- and post-intervention values of rising index, weight-bearing symmetry, and 10MWT scores, while the Wilcoxon signed-rank test was used for weight transfer time and COG sway velocity.

Statistically significant changes were observed between pre- and post-intervention values for STS parameters (weight transfer time, rising index, COG sway velocity, and weight-bearing symmetry) in both groups (p < 0.05), as shown in Table 2.

**Table 2:** Intragroup analysis of sit-to-stand ability test parameters sec: Seconds; %: Percentage; deg/sec: Degrees per second; COG: Center of gravity.

A. Weight Transfer Time (sec)		Mean	Std. Deviation	Mean Difference	P-Value	Significance
Group A	Pre	1.030	0.733	-0.098	0.0021	Significant
	Post	0.932	0.687			
Group B	Pre	0.943	0.534	-0.005	0.0001	Significant
	Post	0.938	0.530			
B. Rising Index (% Body Weight)		Mean	Std. Deviation	Mean Difference	P-Value	Significance
Group A	Pre	13.94	6.68	2.59	0.0026	Significant
	Post	16.53	7.04			
Group B	Pre	13.53	5.85	0.76	0.0076	Significant
	Post	14.29	5.85			
C. COG Sway Velocity (deg/sec)		Mean	Std. Deviation	Mean Difference	P-Value	Significance
Group A	Pre	4.13	1.15	-0.47	0.0020	Significant
	Post	3.66	1.22			
Group B	Pre	3.95	1.16	-0.09	0.0215	Significant
	Post	3.86	1.17			
D. Weight-Bearing Symmetry (%)		Mean	Std. Deviation	Mean Difference	P-Value	Significance
Group A	Pre	16.65	9.35	-2.3	<0.0001	Significant
	Post	14.35	9.03			
Group B	Pre	19.53	7.39	-0.06	<0.0001	Significant
	Post	19.47	7.53			

Similarly, gait speed assessed using the 10MWT demonstrated statistically significant improvement in both groups following the intervention (p < 0.05), as shown in Table 3. For parameters such as weight transfer time, COG sway velocity, and weight-bearing symmetry, a reduction in values indicates improved performance, whereas an increase in the rising index reflects improved lower limb force generation. Weight-bearing symmetry is expressed as a percentage difference between limbs; therefore, negative values represent a reduction in asymmetry and indicate improved symmetry of weight distribution.

**Table 3:** Intragroup analysis of 10-meter walk test scores m/s: Meters per second.

10-Meter Walk Test Scores (m/s)		Mean	Std. Deviation	Mean Difference	P-Value	Significance
Group A	Pre	0.53	0.19	0.09	<0.0001	Significant
	Post	0.62	0.21			
Group B	Pre	0.42	0.09	0.02	0.0025	Significant
	Post	0.44	0.1			

Intergroup analysis

**Figure 3:**
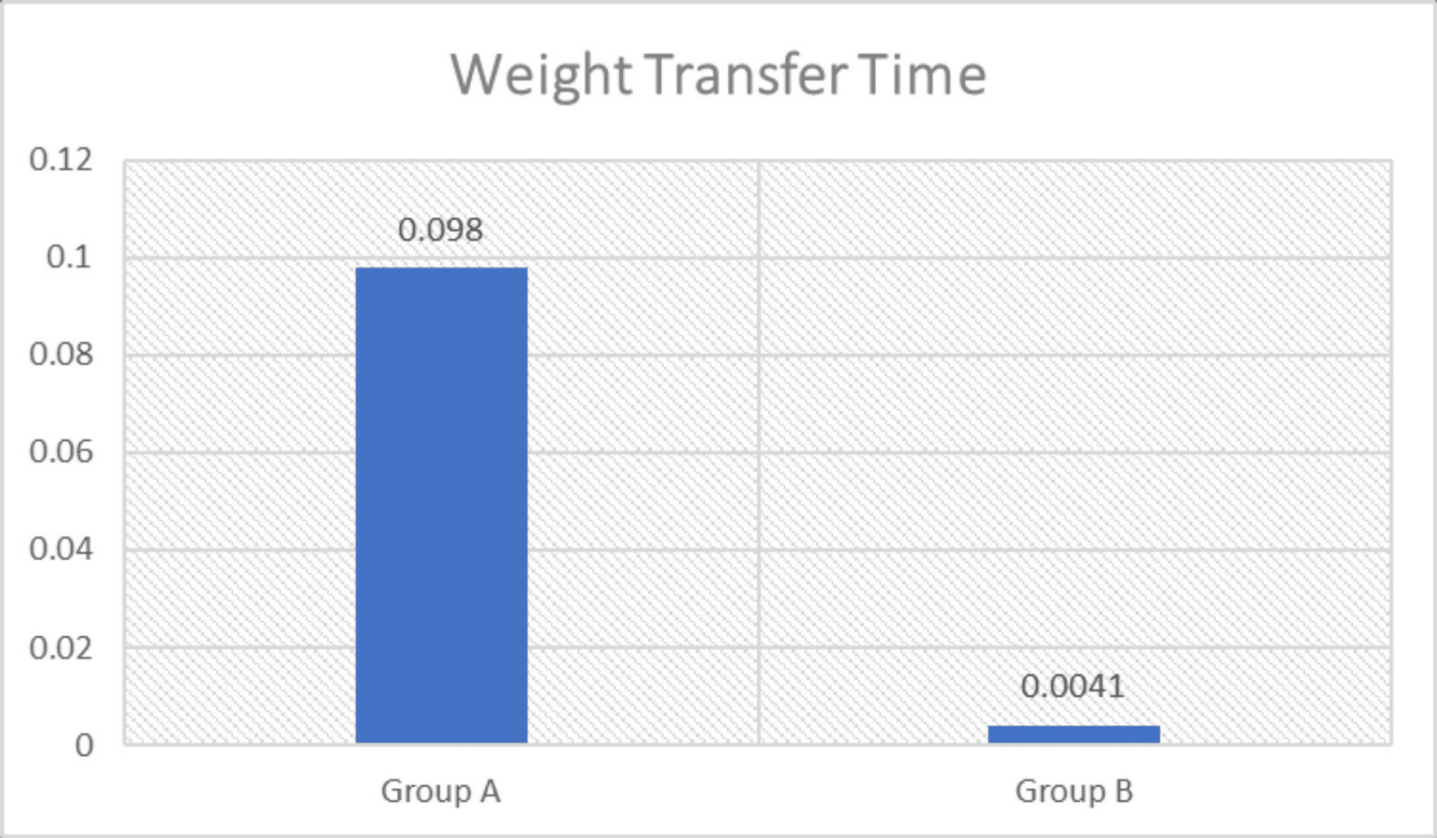
Comparison of weight transfer time (sec) between the groups sec: Seconds.

**Figure 4:**
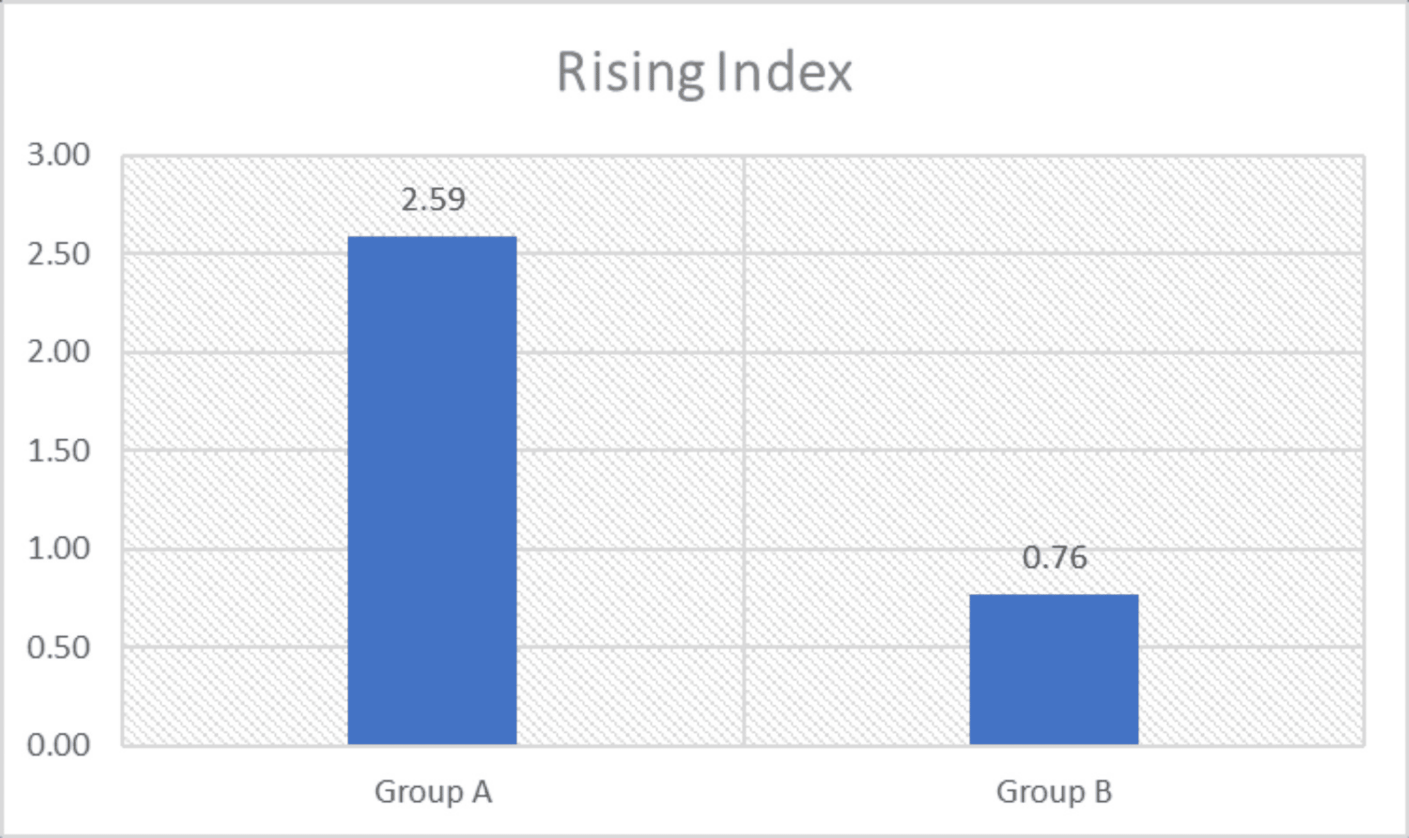
Comparison of rising index (% body weight) between the groups

**Figure 5:**
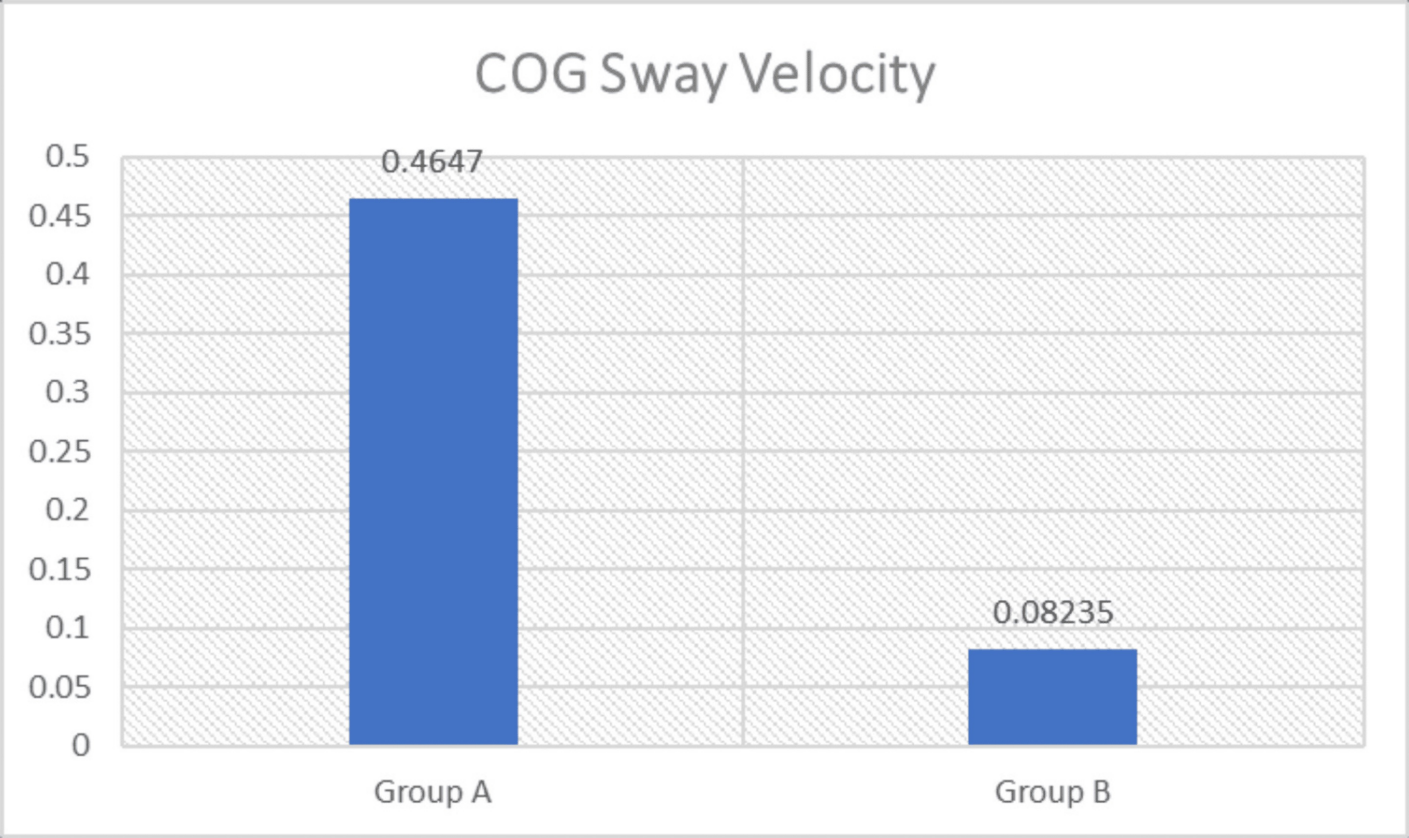
Comparison of COG sway velocity (deg/sec) between the groups COG: Center of gravity; deg/sec: Degrees per second.

**Figure 6:**
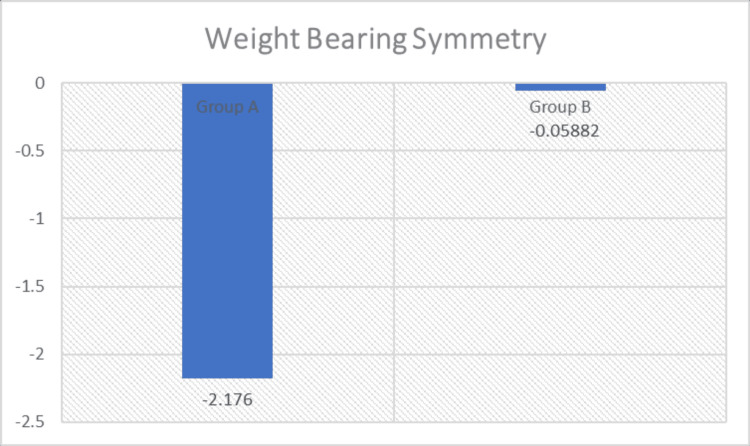
Comparison of weight-bearing symmetry (%) between the groups

Intergroup comparisons were performed using the Mann-Whitney U test to determine differences between Group A (loaded STS exercise combined with conventional therapy) and Group B (conventional therapy). A statistically significant difference was observed between the groups for STS parameters, including weight transfer time, rising index, COG sway velocity, and weight-bearing symmetry (p < 0.05), with greater improvement noted in Group A (Figures 3-6 and Table 4). The magnitude of change was greater in Group A across all parameters, including greater reductions in weight transfer time, COG sway velocity, and weight-bearing asymmetry, along with a greater increase in rising index.

**Table 4:** Intergroup analysis of sit-to-stand ability test parameters sec: Seconds; %: Percentage; deg/sec: Degrees per second; COG: Center of gravity.

A. Weight Transfer Time (sec)	Mean Change (Post–Pre)	Difference in Mean Change (A–B)	Std. Deviation	P-Value	Significance
Group A	-0.098	-0.094	0.17	<0.0001	Significant
Group B	-0.004		0.015		
B. Rising Index (% Body Weight)	Mean Change (Post–Pre)	Difference in Mean Change (A–B)	Std. Deviation	P-Value	Significance
Group A	2.588	1.823	2.575	<0.0001	Significant
Group B	0.765		1.033		
C. COG Sway Velocity (deg/sec)	Mean Change (Post–Pre)	Difference in Mean Change (A–B)	Std. Deviation	P-Value	Significance
Group A	-0.465	-0.383	0.4372	<0.0001	Significant
Group B	-0.082		0.1237		
D. Weight-Bearing Symmetry (%)	Mean Change (Post–Pre)	Difference in Mean Change (A–B)	Std. Deviation	P-Value	Significance
Group A	-2.176	-2.117	0.7276	<0.0001	Significant
Group B	-0.059		0.8993		

**Figure 7:**
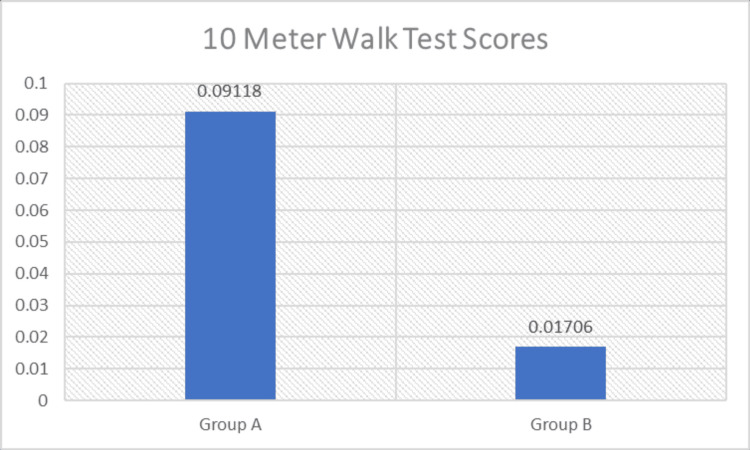
Comparison of 10-meter walk test scores (m/s) between the groups m/s: Meters/second.

Similarly, gait speed measured using the 10MWT demonstrated a statistically significant difference between the two groups (p < 0.05), indicating greater improvement in Group A compared with Group B (Figure 7 and Table 5).

**Table 5:** Intergroup analysis of 10-meter walk test scores (m/s) m/s: Meters per second.

10-Meter Walk Test Scores (m/s)	Mean	Mean Difference	Std. Deviation	P-Value	Significance
Group A	0.09118	0.07142	0.04554	<0.0001	Significant
Group B	0.01706		0.01961		

Overall, the findings indicate that loaded STS exercise combined with conventional therapy resulted in greater improvements in STS ability and gait speed compared with conventional therapy alone.

## Discussion

The present study demonstrated that loaded STS exercise combined with conventional therapy resulted in greater improvements in STS ability and gait speed in individuals with stroke compared to conventional therapy alone. Statistically significant intragroup improvements were observed in STS parameters and gait speed in both Group A and Group B following the intervention.

The statistically significant improvements in STS parameters, including weight transfer time, rising index, COG sway velocity, and weight-bearing symmetry, observed in Group A may be attributed to the addition of external loading during the STS task, which increases the demand on lower limb musculature and enhances neuromuscular activation. Repetitive performance of STS with trunk loading may improve lower limb strength, postural control, and symmetrical weight-bearing, which are commonly impaired following stroke. These findings are consistent with the research conducted by Sahu and Halder, who demonstrated significant improvements in balance and gait speed following a loaded STS exercise intervention [10]. The application of weights around the trunk during the movement may enhance proprioceptive feedback and postural stability, thereby reducing sway during the stabilization phase of the task. Previous studies have also reported that repetitive STS training improves STS ability, lower limb strength, and balance in stroke survivors [15,16]. Additionally, repeated task-specific practice may promote motor learning and improved motor control through neuroplastic mechanisms, supporting the principle of “use it and improve it,” which facilitates adaptive neural changes and improved functional performance [17,18].

Participants in Group B who received conventional therapy also demonstrated statistically significant improvements in STS​​​​​​​ performance. Conventional neurorehabilitation approaches such as PNF, the Bobath approach, and the MRP focus on facilitating normal movement patterns, improving postural control, and enhancing motor recovery. Functional exercises, including bridging, squatting, balance training, and weight-bearing activities, likely contributed to improved neuromuscular coordination and postural stability during STS​​​​​​​ performance. Previous research has shown that task-specific training and repetitive functional exercises can enhance motor recovery and improve balance performance in individuals with stroke [2,19].

Intergroup comparison revealed statistically significantly greater improvements in STS​​​​​​​ parameters in the experimental group compared to the control group. The additional resistance provided through trunk loading may have enhanced recruitment of the hip and knee extensors involved in the STS​​​​​​​ movement while also providing a task-specific strengthening stimulus. In addition, external loading may increase proprioceptive input through joint compression, improving postural feedback and reducing sway [20]. Similar findings were reported by Hyuk Jae Choi, who observed improved stability and reduced sway excursion with torso weighting compared to a non-weighted intervention [21]. Possible mechanisms include joint compression, increased awareness of the weighted segments, and enhanced sensory input to the central nervous system that helps modify motor output [22]. When combined with conventional physiotherapy interventions such as strengthening, functional training, and balance exercises, loaded STS​​​​​​​ training may be associated with greater improvements in functional performance.

Improvements in STS​​​​​​​ performance may also have functional implications for mobility tasks such as walking, as efficient STS​​​​​​​ transitions require adequate lower limb strength, balance control, and symmetrical weight-bearing. Therefore, enhancement of these parameters may subsequently contribute to improvements in gait performance in individuals with stroke. However, the extent to which these improvements translate into clinically meaningful functional outcomes requires further investigation.

The present study also evaluated gait speed using the 10MWT. Both groups demonstrated statistically significant improvements in gait speed following the intervention; however, greater improvement was observed in participants who received loaded STS​​​​​​​ exercise along with conventional therapy. The improvement in gait speed in Group A may be related to increased activation and strengthening of the hip and knee extensors involved in both STS​​​​​​​ transitions and walking. STS​​​​​​​ is a functional task requiring coordinated activation of the quadriceps and gluteal muscles, which play an essential role in propulsion during gait. Repetitive practice with trunk loading may therefore enhance lower limb strength, promote greater weight-bearing on the paretic limb, and improve postural stability, ultimately contributing to improved walking performance. Similar findings were reported by Shin and Lee, who observed improvements in gait ability in stroke patients following gait training using a weighted jacket [23].

Participants in Group B also demonstrated statistically significant improvements in gait speed following conventional therapy. Conventional physiotherapy interventions such as strengthening exercises, balance training, treadmill walking, and functional mobility training likely contributed to these improvements. These interventions aim to improve lower limb strength, coordination, and balance, which are essential components for efficient gait performance. Previous studies have reported that task-specific training, progressive strengthening exercises, and treadmill walking can improve gait parameters such as speed, step length, and cadence in individuals with stroke [3,24].

Intergroup comparison revealed statistically significant greater improvement in gait speed in the experimental group compared to the control group. The additional resistance provided during loaded STS​​​​​​​ training may enhance paretic limb loading and strengthen the hip and knee extensors, thereby improving propulsion during walking. Increased activation of lower limb extensor muscles helps stabilize the stance phase and facilitates forward progression of the body, which may result in improved step length and increased gait speed [3,15].

Overall, the findings of this study suggest that incorporating loaded STS​​​​​​​ exercise into conventional physiotherapy programs may be beneficial for improving STS​​​​​​​ ability and gait speed in individuals with stroke. The improvements observed in functional performance indicate that task-specific strengthening with external loading may enhance lower limb strength, postural control, and symmetrical weight-bearing, thereby contributing to improved mobility and greater functional independence among stroke survivors.

Limitations

Despite these positive findings, the present study was limited by the absence of follow-up assessment; therefore, the long-term retention of improvements could not be determined. The relatively small sample size and gender distribution may also limit generalizability. Additionally, while statistically significant improvements were observed, the clinical relevance of these changes was not specifically evaluated. Future studies with larger sample sizes and follow-up assessments are recommended.

## Conclusions

Based on the findings of the present study, it can be concluded that loaded STS exercise combined with conventional physiotherapy may be effective in improving STS ability and gait speed in individuals with stroke. Participants who received loaded STS training along with conventional therapy demonstrated greater improvements compared to those who received conventional therapy alone.

The addition of external loading during the STS task may enhance lower limb muscle activation, improve postural control, and promote symmetrical weight-bearing, thereby facilitating better functional performance. Improvements in STS ability may also contribute to enhanced gait performance, as both tasks require adequate lower limb strength, balance, and coordinated movement. Therefore, incorporating loaded STS exercise into conventional physiotherapy programs may serve as an effective task-specific intervention for improving functional mobility and promoting greater independence in individuals with stroke.
